# Quality of Life Considerations in Endoscopic Endonasal Management of Anterior Cranial Base Tumors

**DOI:** 10.3390/cancers15010195

**Published:** 2022-12-28

**Authors:** Anirudh Saraswathula, Jose L. Porras, Debraj Mukherjee, Nicholas R. Rowan

**Affiliations:** 1 Department of Otolaryngology-Head and Neck Surgery, Johns Hopkins University School of Medicine, Baltimore, MD 21287, USA; 2 Department of Neurological Surgery, Johns Hopkins University School of Medicine, Baltimore, MD 21287, USA

**Keywords:** quality of life, endoscopic endonasal skull base surgery, rhinology, skull base, cranial base malignancies

## Abstract

**Simple Summary:**

Considering quality of life (QOL) is critical when discussing treatment options for patients undergoing endoscopic endonasal skull base surgery for cancers at the base of the skull. In this review article, we examine the different questionnaires that have been developed to assess QOL for these patients, consider how both tumors themselves and different surgical approaches have been found to affect QOL, as well as look ahead to new and emerging tools and techniques aimed to help preserve and improve QOL in patients with anterior cranial base malignancies.

**Abstract:**

Considering quality of life (QOL) is critical when discussing treatment options for patients undergoing endoscopic endonasal skull base surgery (EESBS) for cancers at the base of the skull. Several questionnaires have been developed and validated in the last 20 years to explore QOL in this patient population, including the Anterior Skull Base Questionnaire, Skull Base Inventory, EESBS Questionnaire, and the Sino-Nasal Outcome Test for Neurosurgery. The Sino-Nasal Outcomes Test-22 and Anterior Skull Base Nasal Inventory-12 are other tools that have been used to measure sinonasal QOL in anterior cranial base surgery. In addition to pathology-related perturbations in QOL endoscopic surgical options (transsellar approaches, anterior cranial base surgery, and various reconstructive techniques) all have unique morbidities and QOL implications that should be considered. Finally, we look ahead to new and emerging techniques and tools aimed to help preserve and improve QOL for patients with anterior cranial base malignancies.

## 1. Introduction

In recent years, patient quality of life (QOL) has become an important part of the conversation in oncology, reflecting broader trends in the medical world. In cranial base oncology in particular, discussions of patient QOL have been more pronounced than ever, especially due to revolutions in surgical approaches. This anatomic region has always been fraught with devastating complications and QOL implications for patients due to the critical neurovascular and aesthetic structures near or involved by tumor. Over the last 30 years, however, with the advent of endoscopic endonasal skull base surgery (EESBS) treatment paradigms for anterior cranial base tumors have dramatically evolved. For select patients and pathologies, this approach has been shown to result in shorter or similar length of stay (LOS) [1], lower complication rates [2], and improved intraoperative exposure, all without sacrificing surgical outcomes [3,4]. Despite some notable advantages posed by EESBS compared to traditional open surgical techniques, skull base surgery still inflicts substantial patient morbidity, and as a result, there is substantial interest in improving QOL outcomes for patients with anterior cranial base pathologies [5]. Additionally, in addition to being a core part of a patient-centered care model [6], QOL is also increasingly used in evaluations of healthcare value such as cost-effectiveness analyses [7].

Herein, we review (1) modern instruments for measuring QOL in patients with cranial base malignancies, (2) important QOL considerations surrounding anterior cranial base malignancies, (3) QOL implications of EESBS approaches used to treat anterior cranial base malignancies, and (4) the future of QOL in cranial base oncology.

## 2. Measuring Quality of Life in Patients with Cranial Base Malignancies

In her foundational work conceptualizing the modern understanding of patient QOL, Bergner lays out five key components of health: the (1) genetic foundation, (2) biological condition, (3) functional condition, (4) mental condition, and (5) health potential [8]. All QOL instruments thus measure some combination of these components, and can be divided into general QOL and anatomic- or condition-specific QOL measures. In cranial base oncology, both site and symptom-specific QOL instruments are highly useful (Table 1), as they focus on elements most germane to treatment discussions and patient counseling. In particular, sinonasal QOL measures are imperative in evaluating perturbations that may result from tumor removal. While tumor removal may alleviate some QOL disruptions, it may also lead to untoward QOL outcomes incurred by manipulation of sinonasal anatomy by using the endoscopic endonasal corridor. Finally, general QOL measures can also be applied and are critical for an all-inclusive evaluation of patient QOL, but these measures are more seldom evaluated in studies of patients with anterior cranial base malignancies.

### 2.1. Cranial Base-Specific Quality of Life Measures

Overall, though multiple general-purpose QOL instruments are available, they are largely underutilized in the cranial base malignancy population [16]. The four most highly used, validated, cranial base-specific QOL measures are the (1) Anterior Skull Base Questionnaire (ASBQ) [9], (2) Skull Base Inventory (SBI) [10], (3) EESBS Questionnaire (EES-Q) [11], and the (4) Sino-Nasal Outcome Test for Neurosurgery (SNOT-NC) [12]. Of these, the ASBQ and the SBI are the more well-known and widely applied.

Published by Gil et al. [9] the ASBQ was the first validated, and widely utilized, cranial base-specific QOL measure. Developed from a cohort of 35 patients with anterior cranial base tumors, the ASBQ includes 35 questions in domains: performance, physical function, vitality, pain, specific symptoms, and influence on emotions. Internal consistency, reliability, and construct validity were demonstrated in the original study, and a later meta-analysis [17] defined a minimal clinically important difference (MCID) of 0.4. While this is the most widely used and cited cranial base-specific QOL measure and was later validated in patients undergoing EESBS [18], it is important to note that the ASBQ does not apply specifically to patients who undergo EESBS approaches to their tumors [19] and was originally designed using surveys of patients undergoing open craniofacial surgery. In fact, only three of the 35 questions in this questionnaire pertain to sinonasal symptoms or function.

Published in 2012 by de Almeida et al., the SBI was the next major cranial-base specific QOL measure to be developed [10]. In contrast to the ASBQ, the initial validation of this instrument included both patients undergoing open surgery and those undergoing EESBS, but the SBI cohort also included patients with central cranial base lesions. This measure is an 11-domain, 41-item questionnaire with 26 disease-specific questions, and later research demonstrated internal consistency and test–retest reliability [20]. More recently, psychometric testing of the instrument has demonstrated good concurrent validity between the SBI and ASBQ as well as moderate correlation with SNOT-22 scores and global health-related-QOL (HR-QOL) measures [21]. The defined MCID for the SBI is 6.0 [21]. Notably, even with this published evidence, the SBI does remain less utilized in the cranial base literature than the ASBQ, which may be due in part to the instrument having only recently undergone psychometric validation.

A newer disease-specific QOL measure in this field, the EES-Q was developed by Dam et al. first published in 2017, [11] with its full psychometric evaluation published in 2019 [22]. Importantly, though patients with cranial base pathology (pituitary adenomas) were included in this initial cohort, it is worth noting that malignant cases were not included, and almost two-thirds of the patient cohort had benign inflammatory sinonasal pathology undergoing endoscopic sinus surgery. Regardless, the EES-Q is likely the simplest of the disease-specific measures discussed here, with three domains (physical, psychological, and social) assessed over 30 questions. Its use and validation, however, is limited to patients undergoing sinus surgery or EESBS, reflected in recent work extending its application to patients with chronic rhinosinusitis (CRS) [23].

### 2.2. Sinonasal Quality of Life Measures in Anterior Cranial Base Surgery

Provided the availability of cranial-base specific QOL measures, there has been tremendous interest in evaluating the QOL of patients undergoing EESBS in the last 10 years [24,25,26,27,28,29,30,31]. Studies evaluating sinonasal QOL, in particular, have proliferated immensely [32,33,34,35,36,37,38,39]. In fact, there has been such an explosion of these studies that there has been discussion of a need for improved and coordinated perioperative data collection in cranial base surgery [40]. The vast majority of these studies, when evaluating sinonasal-specific QOL, utilize one of two measures: the Sino-Nasal Outcomes Test-22 (SNOT-22) or the Anterior Skull Base Nasal Inventory-12 (ASK Nasal-12).

The SNOT-22 is one of the most widely used and validated QOL instruments in otolaryngology. It has undergone significant evolution since it was first developed in the 1990s as the SNOT-20 [41] after removal of 11 items from the Rhinosinusitis Outcome Measure-31 [42] in a bid to improve the instrument’s ease of use while maintaining reliability, validity, and responsiveness. The SNOT-22 was the next step in this measure’s development, with changes in scoring and the addition of questions addressing olfactory function [14,43], and was ultimately used in the National Health Service’s National Comparative Audit of Surgery for Nasal Polyposis and Rhinosinusitis, published in 2006 [44]. It has since been considered the optimal outcome tool in CRS, and has been used extensively both in evaluating sinonasal QOL in EESBS patients [29,32,33,34] and for validation of new cranial-base specific QOL measures [11,13,23]. However, it is important to remember that the SNOT-22 was not formally validated in EESBS and, as recently noted in a cranial base population, does not account for a number of important factors relevant to EESBS patients such as visual, endocrine, and neurological symptoms [45]. Its length, ability to be applied as a single construct only, and the fact that half of its items are not affected by EESBS have also been pointed out as limitations in the cranial base malignancy population [45].

The ASK Nasal-12 measure was developed more recently with its first description in 2013 by Little et al. [15]. The instrument, comprised only of 12 items, was validated and its psychometric properties defined in a population of patients undergoing transsphenoidal surgery for pituitary lesions. This questionnaire is exclusively focused on sinonasal symptoms and has a defined MCID of 0.37, out of a total possible score of 5 [46]. While it is a newer instrument, some authors consider it a useful tool for EESBS patients given its narrower validation cohort and excellent construct validity in this population [47,48].

Published in 2020, the SNOT-NC is the newest cranial-base specific QOL instrument. The SNOT-NC was originally developed and validated in German by Ahmadipour et al. [12] and Italian by Riva et al. [13]. The SNOT-NC was developed from the SNOT-22 instrument and validated with the SF-36, a broad HR-QOL measure, in 102 patients who underwent transsphenoidal pituitary surgery. It assesses five domains (nasal discomfort, sleep problems/reduced productivity, ear and head discomfort, visual impairment, and olfactory disturbance) over 23 items. However, the SNOT-NC is not yet validated in English and has only been validated for patients undergoing EESBS for pituitary lesions thus limiting its more widespread adoption. Despite its limitations, the SNOT-NC is notable amongst cranial-base specific QOL instruments given its strong focus on sinonasal QOL.

## 3. Quality of Life Considerations

### 3.1. Tumor Consequences on Quality of Life

Even prior to treatment, anterior cranial base malignancies can result in significant perturbations of patient QOL, affecting sinonasal QOL, HR-QOL, and global QOL. In fact, these perturbations are often how the tumor is detected and diagnosed, and largely relate to the mass effect the tumor has on adjacent cranial base and sinonasal structures [49]. Vision is commonly impacted due to the proximity of the optic chiasm, the optic nerves, the extraocular muscles, and the globe itself. Advanced sinonasal malignancies can erode through the lamina papyracea into the orbit, and sellar-based tumors can exert pressure on the optic chiasm. The disruption of trigeminal nerve branches emerging from the foramen ovale or the foramen rotundum can affect facial sensation and parasympathetic functions such as tearing. Sinonasal malignancies may extend intracranially leading to morbidities such as damage to frontal lobe structures, cerebrospinal fluid (CSF) leaks, or ascending meningitis [50].

Anterior cranial base malignancies can also lead to significant sinonasal morbidity. Nasal breathing can be impacted by nasal obstruction from a mass. Smell perception can also be affected, especially in tumors involving or obstructing the olfactory cleft in the superior nasal cavity where most of the afferent olfactory fibers course through the olfactory neuroepithelium. Disruptions in the sense of smell may also be associated with changes in the perception of flavor or self-reported taste. Meanwhile, proptosis, skin involvement, or tumor erosion of the nasal septum can cause functional and aesthetic changes as well. Finally, global physical and psychosocial QOL changes can be expected as sequelae from cancer. Cancer can result in fatigue, sleep changes, and significant psychological distress increasing the risk of depression and anxiety [50].

### 3.2. EESBS Approaches and Quality of Life

More commonly discussed, however, is how EESBS approaches affect patient QOL, particularly when compared to open approaches. Numerous studies have also evaluated how cranial base-specific and sinonasal QOL measures are affected by different EESBS approaches and specific anatomic considerations [28,29,30,31,32,33,34,51,52,53,54,55]. While the literature here is vast, the most important conclusion is that, broadly speaking, sinonasal QOL is worse immediately after EESBS due to postoperative changes, but this transient effect typically resolves within several months. Meanwhile, cranial-base specific QOL is largely comparable or improved with EESBS approaches in select patients. Many of these studies, however, are limited by patient and disease heterogeneity, lack of standardization in QOL instruments, and limited study size, an impediment to some of the meta-analyses that have been performed [17,35,55].

Perhaps the most common sinonasal symptom many of the studies, particularly the earliest ones, have focused on, is nasal crusting and drainage. In early experiences, crusting lasted about four months [29,30], on average, and was present in almost all patients [38], though other studies have shown rates as low as 36% [56]. Other complications including nasal synechiae, hypesthesia, and taste disturbances were far less common. Nasal breathing and obstruction have also been evaluated extensively. In one study, over 90% of patients report no changes in nasal airflow one month following EESBS [38]. These findings have been replicated broadly [32,33,34,35,36,37,38,39], though admittedly the vast majority of patients in these studies have pituitary pathology.

Interestingly, many of these sinonasal symptoms are short-lived and likely related to the postoperative healing process in the endonasal corridor. In a 2015 review of 81 patients undergoing EESBS, McCoul et al. described transient worsening of sinonasal (SNOT-22) and cranial-base specific QOL (ASBQ) at three weeks postoperatively, but at six months and one year after surgery, sinonasal QOL was unchanged and cranial-base specific QOL was improved on average. They also noted that subtotal resection was associated with lower QOL [53]. This observation was again demonstrated in a large 2019 meta-analysis [55] of studies [34,51,53,57,58,59,60,61,62,63,64,65,66] examining sinonasal QOL using the SNOT-22 instrument in patients undergoing EESBS. This study found that for those patients with impaired sinonasal QOL preoperatively, EESBS improved their sinonasal QOL on average. Patients with normal preoperative sinonasal QOL had no significant change in SNOT-22 scores ≥ 96 weeks after surgery [55]. They additionally evaluated sinonasal QOL in patients with intranasal versus intracranial pathology and found that those with preoperative intranasal pathology had worse baseline sinonasal QOL than those with intracranial disease. Both intranasal and intracranial groups, however, showed similar trajectories in postoperative sinonasal QOL until one year postoperatively, when sinonasal QOL was worse for those with intranasal pathology. The authors hypothesized that this was possibly sequelae secondary to sinonasal scarring or an improved emotional state of the intracranial pathology group.

Recently, more attention has been given to olfaction as a subdomain of sinonasal QOL, particularly in trying to preserve it with procedural alterations. Using the University of Pennsylvania Smell Identification Test (UPSIT), Sowerby et al. found no postoperative difference in smell within a cohort of 21 EESBS patients whose middle turbinates were sacrificed [67], nor did Raikundalia et al. in a study that administered the UPSIT and the Assessment of Self-reported Olfactory Functioning in patients undergoing transsphenoidal surgery for benign sellar lesions [33]. In a cohort of 14 patients with esthesioneuroblastoma undergoing postoperative radiation, who typically sustain complete postoperative anosmia, Tajudeen et al. found that when preserving one olfactory bulb, 43% of their patients had residual olfactory function, and 14% had normal or mildly reduced function [68]. Other studies have commented on preserving the so called “olfactory strip” of the superior septal mucosa when oncologically possible, by either using a nasoseptal flap for cranial base reconstruction, using a smaller amount of mucosal tissue thus sparing the olfactory epithelium [36,51], or using a cold knife instead of cautery to incise the mucosa [61]. While a large meta-analysis of 29 studies measuring olfactory outcomes in EESBS showed no difference in preoperative and postoperative olfaction, the authors commented that the study was limited by a need for more objective and standardized assessments of smell [35].

Studies comparing open surgery to EESBS have been limited due to challenges with selection bias, however, one relatively well-matched study from 2012 showed clinically important differences in the ABSQ’s physical function and emotional domains. Those undergoing EESBS appeared to have improved disease-specific QOL in this analysis [69].

#### 3.2.1. Sella and Parasellar Area

As mentioned above, numerous studies have shown that sinonasal QOL returns to baseline or near baseline in most patients undergoing pituitary EESBS. McCoul et al. [53] showed this and was supported by Little et al. [48] in a post hoc analysis of data from a multicenter study of EESBS patients with pituitary lesions. Suberman et al. [70] and Wu et al. [71] have also supported these findings in patients with sellar lesions, and the Wu study additionally noted that SNOT-22 scores were not associated with intraoperative CSF leak repair, tumor pathology, or a history of nasal surgeries. While most studies in this realm evaluate patients with pituitary tumors, the QOL of patients with less common anterior cranial base pathologies has also been investigated. Patel et al. administered the ABSQ and the SNOT-22 to 31 patients undergoing EESBS for craniopharyngiomas. Unlike studies in pituitary lesion patients, this cohort did not show a significant change in cranial-base or sinonasal specific QOL over nine months postoperatively, and authors noted that these patients had lower postoperative ABSQ scores on average than a cohort undergoing EESBS for resection of pituitary macroadenoma. Interestingly, better QOL was associated with a gross total resection of the mass and receipt of postoperative radiation therapy [72]. It is worth noting, however, that very few patients with sellar or parasellar pathology have malignant pathology, and much more likely to have benign lesions and so some data may be challenging to extrapolate.

#### 3.2.2. Anterior Cranial Fossa

Other anterior cranial fossa lesions, particularly those abutting or involving the cribriform plate of the ethmoid bone (e.g., esthesioneuroblastomas, meningiomas, and sinonasal cancers), may also be managed endoscopically. Historically, skull base reconstruction was a much greater concern in these patients, but this has significantly improved since the development of the Hadad-Bassagasteguy nasoseptal flap [73]. These patients are certainly also affected by many of the same concerns that patients with sellar or parasellar lesions have, but their sinonasal QOL does not return to baseline as we see in patients with pituitary lesions [74]. This could potentially be driven by many of these patients requiring postoperative adjuvant chemoradiation, causing ongoing local tissue damage and remodeling, Data on QOL for patients undergoing EESBS for other anterior cranial fossa lesions is mixed. In a cohort of patients undergoing EESBS for anterior cranial base meningiomas, Jones et al. [59] also saw no difference pre- and postoperatively in ABSQ scores, but they found that SNOT-22 scores did improve in the long-term postoperative period. Interestingly, cranial-base and sinonasal specific QOL was lower in meningioma patients older than 55 years. Finally, Choi et al., in a subgroup analysis of patients who undergoing surgery for extrasellar pathologies (i.e., patients undergoing transpterygoid, transcribriform, transclival, or transplanum approaches instead), did not show diminishment in sinonasal QOL from baseline [39].

In this region, olfaction represents a specific concern because of the proximity of these lesions to the olfactory mucosa near the cribriform plate. In fact, a systematic review by Purohit et al. found that over 95% of esthesioneuroblastoma patients undergoing EESBS suffered from postoperative anosmia compared with 37% of those treated with transcranial surgery [75]. With this concern in mind, olfaction-preservation is a consideration (far behind oncologic resection), which is what motivated the previously mentioned 2016 Tajudeen et al. study [68] demonstrating endoscopic unilateral resection of esthesioneuroblastoma in carefully selected cases (following by adjuvant chemoradiation). Forty percent of the patients in this cohort had residual smell function, with no disease recurrence with a mean follow up of 51.7 months.

#### 3.2.3. Reconstruction

There are several reconstructive options for cranial base defects after EESBS, ranging from the most common (free mucosal grafts or nasoseptal flaps) to less routine vascularized flaps such as inferior turbinate flaps or tunneled temporoparietal fascia flaps. Pericranial flaps and free flaps are more heavily utilized in open craniofacial surgery. Several studies have investigated whether the choice of reconstructive technique influences patients’ postoperative QOL. Interestingly, one of the larger studies in this realm did not see a major difference in QOL based on extrasellar tumor extension, intraoperative CSF leakage, or reconstruction technique, though this was a study in patients with benign pathology [53]. This was supported by a study that demonstrated patient-reported experience of nasal morbidity from a nasoseptal flap correlated poorly with physician endoscopic exam during the healing process [76]. Other research [77], including a systematic review on the subject [78], largely supports this idea that nasoseptal flaps do not appear to affect postoperative sinonasal QOL [58]. One Australian prospective cohort study showed decreased SNOT-22-measured sinonasal QOL in their subgroup who required a nasoseptal flap [79], as nasoseptal flaps present a variety of well-known complications such as crusting, flap failure, and septal perforation, occurring in 27% of patients in a large retrospective review [63,80]. In fact, prolonged crusting along the cranial base has been noted in up to 5% of patients undergoing vascularized flap for CSF leak repair [81]. Finally, while the nasoseptal flap has revolutionized skull base reconstruction, more recent commentary has suggested that larger flaps in younger patients with malignant pathology may be associated with a higher risk of septal necrosis and subsequent nasal dorsum collapse requiring further corrective surgery [82,83]. This important element of QOL should be considered when counseling patients for EESBS that will require cranial base reconstruction.

## 4. Future Work in Quality of Life for Cranial Base Malignancy Patients

### 4.1. Depression and Anxiety

**Figure 1:**
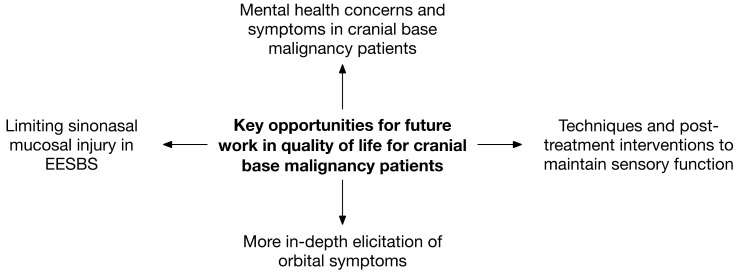
Major opportunities for future work in quality-of-life considerations for patients with anterior cranial base malignancies.

Comorbid mental health concerns in patients with head and neck cancers have been well documented, often due to loss of voice and risk of cosmetic alterations in addition to the stress of a cancer diagnosis. Head and neck cancer patients are among the highest risk in the oncology population for suicide and self-harm [84], a problem unfortunately exacerbated by the COVID-19 pandemic [85]. Even for patients with cranial base malignancies, this is a major issue. A large claims-based study found that the prevalence of mental health disorders increases following diagnosis of a sinonasal/skull base malignancy [86]. Interestingly, in a very small prospective study of patients undergoing EESBS or open cranial base surgery, postoperative depression and anxiety scores were shown to be lower in patients undergoing EESBS [87], though this could certainly be due to a reduced extent of surgery, lack of external incisions, and resultant postoperative sequelae. A prominent randomized controlled trial by Lydiatt et al. in 2013 showed that the initiation of escitalopram therapy in nondepressed patients with head and neck cancer prior to entering cancer treatment reduced the risk of developing depression by over 50% [88]. Given the number of QOL perturbations and mental health risks that cranial base tumors present patients, this could be a valuable line of inquiry in this field with the potential for significant patient benefit (Figure 1).

### 4.2. Sensory Function after EESBS

As discussed above, olfactory dysfunction after EESBS has been extensively studied [55], but only recently have there been inquiries into techniques and interventions to improve olfactory function in this patient population. As mentioned previously, Tajudeen et al. [68] showed that properly selected esthesioneuroblastoma patients can have unilateral, smell-sparing surgery. Medical options are being explored as well. In a randomized study, Yan et al. administered omega-3 supplementation to EESBS patients with persistent olfactory dysfunction due to its effects on wound healing and nerve regeneration. They found that patients receiving the supplementation returned to baseline smell function at six months postoperatively, while those in the control group had persistent olfactory impairment [89]. A limitation of this study may be that most patients with pituitary surgery do not have olfactory loss at six months, but their control cohort unusually had several with persistent olfactory dysfunction.

Loss of smell sense, in addition to impairing olfaction, also affects taste sensation and the flavor and perception of food. The literature on taste sequelae after EESBS for patients with cranial base lesions is far less developed, however. Bedrosian et al. was the first work, in a prospective cohort study using the ASBQ, to report gustatory alterations after EESBS independent of changes in olfaction [90]. The sensation of “taste”, as conceptualized on many questionnaires, is complex, and while the sense is informed considerably by olfactory information, gustatory and somatosensory information is critical as well. However, taste data is limited in EESBS patients, particularly because it comprises only one question on most questionnaires and because reliable objective taste testing is not routinely available. However, in patients undergoing chemotherapy, poor taste function has been shown to be associated with worse nutritional behavior during and after therapy [91], a critical consideration in the surgical population for postoperative healing and for global QOL [92]. Objective gustatory testing has been developed and available for the past 10–15 years, with “Taste Strips”, a bedside test validated in normal participants with flavors impregnated at various concentrations into strips of filter paper that asks participants to distinguish between four flavors: sweet, sour, salty, and bitter [93]. Given the importance of this element of disease-specific QOL in EESBS patients and the fact that taste training may even improve taste function and nutrition in some oncology patients [91], gustatory function could be a promising avenue of further work [94].

Finally, it is worth mentioning the effects on vision as well as the functions of the vidian nerve, palatine nerve, and V2. While some of the cranial-base QOL measures do address vision, there is a dearth of questionnaire items addressing orbital symptoms. Some QOL work has been done in this space, but largely in patients undergoing orbital exenterations [95,96]. This could represent another valuable area of investigation. Injury to or sacrifice of the vidian nerve, palatine nerve, or V2, can occur during transpterygoid or other approaches involving the pterygopalatine fossa. While the sequelae of dry eye, paresthesia, or numbness can be bothersome and noticeable long-term in a small number of these patients [97], the majority of these patients report the consequences of injury to these nerves as “not bothersome” or “mildly bothersome” [98]. Regardless, this may be another area to expand the reach of current QOL instruments.

### 4.3. Mucosal Trauma and Scarring

Many of the important sinonasal QOL sequelae of EESBS, such as crusting, nasal drainage, and resultant nasal obstruction, can be exacerbated by intraoperative mucosal trauma. Sinonasal mucosal preservation is of course a key facet of endoscopic sinus surgery [99], and more recent work has tried to consider modifications of surgical technique to minimize this during EESBS. Reverse rotation flaps have been described, utilizing the contralateral septal mucosa previously covering bone removed during a posterior septectomy to cover and protect exposed cartilage left after the raising of a nasoseptal flap [100]. Another study randomized patients undergoing EESBS with nasoseptal flaps to receive porcine small intestine submucosal grafts or no graft, finding that patients in the experimental arm showed significantly improved remucosalization and with a trend towards less crusting at three months postoperatively [101]. This area of investigation may come to inform modifications to the practice of EESBS in coming years.

## 5. Conclusions

In conclusion, QOL is a critical topic for the treatment of patients undergoing endoscopic endonasal skull base surgery for anterior cranial base malignancies. There is a variety of validated tools to measure quality of life, both sinonasal- and cranial base-specific, each with its own strengths and weaknesses. While the ASBQ is the most widely used, newer instruments may benefit from more substantial and rigorous validation efforts and therefore be able to better inform patients and direct their care. Both cranial base malignancies in and of themselves as well as the surgical approaches used to treat them result in considerable perturbations to QOL, though a large body of work shows that sinonasal QOL returns to baseline in the vast majority of patients after EESBS. However, as we seek to advance the field there is considerable work to be done in standardizing QOL measurement in this patient population and using these data to inform how we counsel patients. Much of what has been done so far has been validated in patients with benign pathology, and there is a wealth of opportunity in this space to develop and hone these tools for patients with malignancies. Finally, new surgical techniques and training tools are also emerging that could present novel ways to help preserve and improve the QOL for patients with anterior cranial base malignancies.

## Figures and Tables

**Table 1:** Anterior cranial base and sinonasal-specific quality of life (QOL) instruments.

Instrument	Focus	Population	Surgical Approach	No. of Items	Questionnaire Type	Domains	Notes
Anterior Skull Base Questionnaire (ASBQ) [9]	Anterior cranial base-specific QOL	Malignant lesions of anterior cranial base	Open	35	Likert (5-point scale)	Performance, physical function, vitality, pain, specific symptoms, influence on emotions	Most widely used Originally defined in patients undergoing open surgery, but later validated in endoscopic candidates
Skull Base Inventory (SBI) [10]		Malignant and benign lesions of anterior and central cranial base	Open and endoscopic	41	Likert (7-point scale)	Social, emotional, physical, cognitive, family, financial, spiritual, endocrine, nasal, neurologic, visual	Originally defined for both open and endoscopic approaches Not as broadly used
Endoscopic Endonasal Sinusand Skull Base Surgery Questionnaire (EES-Q) [11]		Malignant and benign lesions of anterior cranial base and paranasal sinuses	Endoscopic endonasal	30	Likert (5-point scale)	Physical, psychosocial, social functioning	Easier to use than others New, not broadly used Limited data in patients with malignant pathology (only 5% of original study)
Sino-Nasal Outcomes Test for Neurosurgery (SNOT-NC) [12]		Benign lesions of anterior cranial base	Endoscopic endonasal	23	Likert (5-point scale)	Nasal discomfort, sleep problems and reduced productivity, ear and head discomfort, visual impairment, and olfactory disturbance	Only available in German [12] and Italian [13] New, not broadly used Not validated in patients with malignant pathology
Sino-Nasal Outcomes Test-22 (SNOT-22) [14]	Sinonasal QOL	Chronic rhinosinusitis	N/A	22	Likert (5-point scale)	Nasal, ear/facial, sleep, function, and emotion	Not validated in patients with cranial base pathology
Anterior Skull Base Nasal Inventory-12 (ASK Nasal-12) [15]		Pituitary lesions	Endoscopic endonasal	12	Likert (6-point scale)	None yet	No subdomain analysis yet Limited content validation thus far
